# Amplicon sequencing allows differential quantification of closely related parasite species: an example from rodent Coccidia (*Eimeria*)

**DOI:** 10.1186/s13071-023-05800-6

**Published:** 2023-06-17

**Authors:** Susana C. M. Ferreira, Víctor Hugo Jarquín-Díaz, Emanuel Heitlinger

**Affiliations:** 1 Division of Computational Systems Biology, Center for Microbiology and Ecological Systems Science, University of Vienna, Djerassipl. 1, 1030 Vienna, Austria; 2 Institute for Biology. Department of Molecular Parasitology, Humboldt-Universität zu Berlin (HU), Philippstr. 13, Haus 14, 10115 Berlin, Germany; 3 Leibniz-Institut Für Zoo- Und Wildtierforschung (IZW) im Forschungsverbund Berlin E.V., Alfred-Kowalke-Straße 17, 10315 Berlin, Germany; 4 Experimental and Clinical Research Center, a cooperation between the Max-Delbrück-Center for Molecular Medicine in the Helmholtz Association and the Charité - Universitätsmedizin Berlin, Berlin, Germany; 5 Experimental and Clinical Research Center, Charité – Universitätsmedizin Berlin, corporate member of Freie Universität Berlin and Humboldt-Universität Zu Berlin, Lindenberger Weg 80, 13125 Berlin, Germany; 6 Max-Delbrück-Center for Molecular Medicine in the Helmholtz Association (MDC), Berlin, Germany

**Keywords:** *Eimeria*, Parasite infection, Microbiome, Parasite quantification, Amplicon sequencing, qPCR, Body condition

## Abstract

**Background:**

Quantifying infection intensity is a common goal in parasitological studies. We have previously shown that the amount of parasite DNA in faecal samples can be a biologically meaningful measure of infection intensity, even if it does not agree well with complementary counts of transmission stages (oocysts in the case of Coccidia). Parasite DNA can be quantified at relatively high throughput using quantitative polymerase chain reaction (qPCR), but amplification needs a high specificity and does not simultaneously distinguish between parasite species. Counting of amplified sequence variants (ASVs) from high-throughput marker gene sequencing using a relatively universal primer pair has the potential to distinguish between closely related co-infecting taxa and to uncover the community diversity, thus being both more specific and more open-ended.

**Methods:**

We here compare qPCR to the sequencing-based amplification using standard PCR and a microfluidics-based PCR to quantify the unicellular parasite *Eimeria* in experimentally infected mice. We use multiple amplicons to differentially quantify *Eimeria* spp. in a natural house mouse population.

**Results:**

We show that sequencing-based quantification has high accuracy. Using a combination of phylogenetic analysis and the co-occurrence network, we distinguish three *Eimeria* species in naturally infected mice based on multiple marker regions and genes. We investigate geographical and host-related effects on *Eimeria* spp. community composition and find, as expected, prevalence to be largely explained by sampling locality (farm). Controlling for this effect, the novel approach allowed us to find body condition of mice to be negatively associated with *Eimeria* spp. abundance.

**Conclusions:**

We conclude that amplicon sequencing provides the underused potential for species distinction and simultaneous quantification of parasites in faecal material. The method allowed us to detect a negative effect of *Eimeria* infection on the body condition of mice in the natural environment.

**Graphical Abstract:**

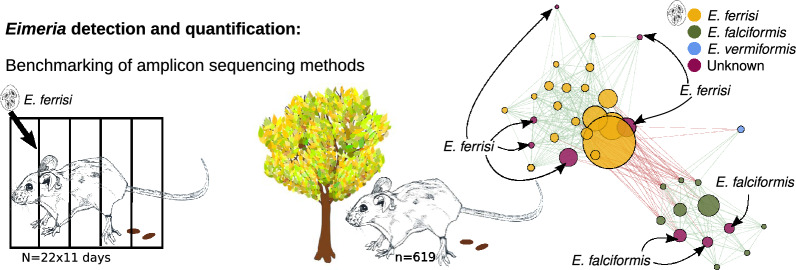

**Supplementary Information:**

The online version contains supplementary material available at 10.1186/s13071-023-05800-6.

## Background

Amplification and sequencing of marker gene fragments, termed “amplicon sequencing”, is widely used in studies of the bacterial microbiome [1]. Similarly, in biodiversity assessment, amplicon sequencing is used to study the biodiversity of eukaryotes [2, 3]. Commonly applied to bacteria in intestinal systems and eukaryotes in terrestrial and aquatic systems, it is surprising how rarely amplicon sequencing is used for intestinal parasites. Amplicon sequencing identified *Eimeria* species in chickens [4, 5] and in other livestock and wildlife [6], but fewer studies have simultaneously estimated parasite abundance from the same data (but see [7–9] for other parasites). While a main focus of bacterial microbiome studies has been on intestinal “ecosystems” within human and animal hosts, sequencing of symbiotic eukaryotes from intestinal contents or faecal samples is less frequently used for detection of (e.g. parasite) taxa and simultaneous differential quantification (assessment of the abundance of multiple species at once).

The intensity of infection with intestinal parasites is classically estimated by counting parasite (transmission) stages present in the faeces. Although automation has progressed [10, 11], classical “coprological methods” are laborious, making it difficult to quantify parasites in high numbers of samples. They also require high technical and taxonomic expertise to distinguish between closely related species. This means that for samples from natural populations, in which co-infections, often with closely related species, are common, differential quantification of multiple species is almost impossible. For this reason, quantitative polymerase chain reaction (qPCR) has become increasingly popular for the quantification of helminths [12, 13] and unicellular parasites, such as Coccidia [14]. DNA-based quantification often shows a considerable discrepancy from counting of transmission stages, but both measures can be complementary in their biological meaning for the host-parasite system, as recently shown for Coccidia [15]. Quantitative PCR, however, requires primer pairs specific to the taxon of interest meaning that quantification has to be targeted and is not easily possible for multiple parasite species in a differential manner.

Multiple potential methodological issues might deter parasitologists from embracing amplicon sequencing for parasite quantification. Universal primer pairs for eukaryotic parasites are not commonly agreed on, in contrast to well-established primers and marker fragments for bacterial microbiome studies [16]. While the ribosomal RNA (rRNA) cluster (18S and 28S ribosomal subunits) in eukaryotes provides suitable targets [17], those have not been validated against the eukaryotic microbiome and parasites. The testing of multiple amplicons might thus be necessary to help the establishment of universal primers for the simultaneous detection of a broad range of taxa. Microfluidic PCR systems compartmentalise and parallelise PCRs in tiny volumes and have been proposed as suitable for easing the work with multiple amplicons [18, 19]. Universal amplification of many parasite taxa not only allows simultaneous differential quantification of multiple (e.g. closely related) target taxa, but is also necessary to provide a background, against which “normalisation” estimates relative abundance. Again, it might be necessary to test multiple approaches for normalisation to optimise quantification with regard to the aims of a study [20–22]. Taxonomic annotation, with the identification of morphologically described species as an ideal commonly aspired, provides the final set of challenges. While modern analysis approaches identify probabilistically likely “amplified sequence variants” (ASVs) [23], their annotation with species names (multiple ASVs might be presenting different species, within species diversity or residual sequencing error) is hampered by imperfect representation of correctly annotated reference sequences in databases [24, 25]. These challenges might seem daunting, but they can be addressed for parasites by careful evaluation of amplicon sequencing against more established techniques.

We here work with *Eimeria* species infecting the house mouse to assess the viability of an amplicon sequencing approach for differential quantification. Three species of *Eimeria* naturally infect the house mouse: *Eimeria ferrisi*, *E. falciformis* and *E. vermiformis* [26, 27]. All species of the genus have a direct life cycle with a predictable genetically determined progression [28]. *Eimeria* spp. have a relatively high prevalence of around 30% (depending on diagnostic method) in natural populations of mice and co-infections have been reported [27]. Laboratory infections of mice with *Eimeria* can easily be conducted and monitored for individual animals, which show a self-limiting infection [15, 29, 30] causing diarrhoea, lack of appetite and weight loss as quantifiable effects [31]. These pathogenic effects are stronger for *E. falciformis* than for *E. ferrisi* [32, 33]. In natural infections, such differences have so far not been detectable.

In this study we used an experimental infection of mice with *E. ferrisi* to benchmark the accuracy of detection and precision of sequencing-based quantification against qPCR. We test whether an amplification in a microfluidics-based device provides a similar quality of quantification to standard PCR and use multiple amplicons in mice sampled in the natural environment to compare marker regions for their taxonomic resolution in very closely related *Eimeria* species. We showcase the methods we established in an assessment of *Eimeria* epidemiology in mice in the natural environment.

## Methods

### Study design

We evaluate the accuracy and usefulness of amplicon sequencing-based parasite quantification in two settings. Firstly, we use laboratory infections of house mice with *E. ferrisi* to establish sensitivity, specificity and quantitative precision of the method against qPCR measurements. The underlying laboratory infection experiments presented here are the same as those used in our previous work comparing qPCR and classical oocyst counting [15]. Secondly, we test differential quantification in mice naturally infected with three different *Eimeria* species. The samples from the natural environment are largely the same as those we analysed in previous work [27, 30]. Not all samples were available for each method, as some mice had not shed sufficient faeces the quantity of extracted DNA not was in some cases not sufficient for all methods. In a few samples, other issues arose during the preprocessing of the samples. We report sample sizes for each analysis.

### Animal husbandry

We obtained in total 22 house mice (*Mus musculus*) of the “wild-derived” inbred strains SCHUNT, STRA, BUSNA and PWD as well as F1 inter-strain crosses [34] from the Institute of Vertebrate Biology of the Czech Academy of Science in Studenec (licence: 61974/2017-MZE-17214). We acclimatised mice to the animal experiment facilities of Humboldt University for at least 1 week before infection. We housed mice in individual cages equipped with tunnels and bedding material for behavioural enrichment and provided them with food and water ad libitum during the experiment.

### Parasite inoculum and experimental infection

For infection, we used the Brandenburg64 isolate of *E. ferrisi*, which had been isolated from the faeces of a wild *M. musculus domesticus* mouse captured in Brandenburg, Germany in 2016 and identified by microscopical description and molecular amplification of the 18S rRNA and cytochrome* c* oxidase (*COI*) markers [27]. We obtained oocysts by continuous passage in NMRI (Naval Medical Research Institute) mice and sporulated them as described previously [33]. We infected mice orally with 150 sporulated oocysts in 100 µl of phosphate-buffered saline (PBS 1×, pH 7.4) and monitored them for 11 days. We recorded the weight daily, and a weight loss of 18% was defined as the humane endpoint at which animals had to be sacrificed (experiment license: 0431/17). At the end of the experiment, we euthanised mice (for which the humane endpoint had not been reached before) by cervical dislocation. We collected an average of 0.12 g of faeces (3–4 faecal pellets) from individual mice daily and stored it after flash-freezing in liquid nitrogen at −80 °C until extraction of DNA.

### Sampling of mice in the natural environment

We trapped 672 mice using live traps in 182 farms or houses between 2015 and 2018. The study area ranges from 51.68° to 53.29° latitude (a 200-km-wide area) and from 12.52° to 14.32° longitude (a 140-km-long area). Each year mice were trapped in September to reduce potential seasonal variation. A median of two mice per locality were captured. Mice were individually isolated in cages and euthanised by cervical dislocation within 24 h after capture (animal experiment permit no. 2347/35/2014). Individual mice were measured (body length from nose to anus), weighed and dissected. Faecal pellets were collected from the cages the mice had been housed in overnight and stored at −80 °C after shock-freezing in liquid nitrogen.

### DNA extraction

We extracted genomic DNA (gDNA) from faeces collected in the infection experiment and in the wild using the NucleoSpin^®^Soil kit (Macherey–Nagel GmbH & Co. KG, Düren, Germany) following the manufacturer’s protocol with the following modifications: we performed mechanical lysis of the sample in the Precellys^®^24 high-speed benchtop homogeniser (Bertin Technologies, Aix-en-Provence, France) using two cycles of disruption at 6000 rpm for 30 s, with 15-s delay between cycles. For each sample, we repeated extraction once to maximise the DNA yield, and nucleic acids were eluted with 40 µl of TE buffer. We assessed the quality and integrity of the DNA using a full-spectrum spectrophotometer (NanoDrop 2000c; Thermo Fisher Scientific, Waltham, MA USA). We quantified concentrations of double-stranded DNA (dsDNA) using a Qubit^®^ Fluorometer and the dsDNA BR (broad-range) Assay Kit (Thermo Fisher Scientific). We adjusted DNA extracts to a final concentration of 50 ng/µl with nuclease-free water (Carl Roth GmbH & Co. KG) and stored them at −80 °C until further processing.

### Real-time qPCR

As a reference measurement, we quantified *Eimeria* DNA by qPCR amplification of a 140-base-pair (bp) fragment of the mitochondrially encoded COI gene (*COI*) using *Eimeria*-specific primers Eim_COI_qX_F 5′-TGTCTATTCACTTGGGCTATTGT-3′ and Eim_COI_qX_R 5′-GGA TCACCGTTAAATGAGGCA-3′. Each reaction contained 1× iTaq™ Universal SYBR^®^ Green Supermix (Bio-Rad Laboratories, Hercules, CA, USA), 400 nM forward and reverse primers and 50 ng template gDNA in a total reaction volume of 20 µl. We performed the reactions in either the ABI 7300 Real-Time PCR System (Applied Biosystems, Thermo Fisher Scientific, Foster City, CA, USA) or the MasterCycler^®^ RealPlex2 machine (Eppendorf, Hamburg, Germany). For both PCR systems, cycling conditions consisted of an initial denaturation at 95 °C for 2 min; 40 cycles of denaturation at 95 °C for 15 s, annealing at 55 °C for 15 s and extension at 68 °C for 20 s. Data were collected at the end of each cycle. Melting curve analysis was included to discard primer dimer formation and non-specific amplification: after the last amplification cycle, the temperature was increased from 65 °C to 95 °C with 0.5 °C increments and 3 s per step. We performed amplifications in triplicate, and each run included a non-template control (NTC). We analysed melting curves blindly for the presence of distinct “*Eimeria* products” and PCR artefacts. We labelled samples with all three replicates showing the melting temperature (T_m_) in the range of 74.1 °C ± 1.78 °C (observed for positive controls) as “qPCR-positive”, samples with only one or two of the triplicates showing a correct peak, were designated as negative samples. For qPCR-negative samples, we set the estimated DNA quantity to zero. For positive samples, we predicted genome copies from a linear model fitted for a standard curve obtained from a known number of oocysts [15]. We adjusted these predictions by dividing them by the amount of starting material the respective DNA was extracted from to obtain an estimate of “genome copies per gram of faeces”.

### Library preparation and sequencing

In total, 236 faecal DNA preparations from the laboratory experiment and 672 from mice sampled in the natural environment were used for a multimarker amplification using the microfluidics-based PCR system Fluidigm Access Array 48 × 48 (Fluidigm, San Francisco, CA, USA). Samples were randomised in their order and amplified in parallel with non-template negative controls using a microfluidic PCR. This makes it possible to amplify multiple fragments (amplicons) for different marker genes (primer pairs in Additional file 1). In an alternative approach, for validation of the microfluidic amplification and sequencing of multiple amplicons, 210 laboratory samples were amplified using the primer pair 5′-GAATTGACGGAAGGGCACC-3′ and 5′-AAGGGCATCACAGACCTGTTAT-3′, targeting the V6-V7 region of the 18S rRNA gene, in standard reaction volumes (96 well microtiter plates). This primer pair has been previously tested to amplify gastrointestinal eukaryotes and showed good coverage of Apicomplexa [9].

For both PCR setups library preparation was integrated into the amplification procedure and was performed according to the protocol Access Array Barcode Library for Illumina Sequencers (single direction indexing) as described by the manufacturer (Fluidigm, San Francisco, CA, USA). The amplicons were quantified (Qubit fluorometric quantification dsDNA High Sensitivity Kit, Thermo Fisher Scientific, Waltham, MA, USA) and pooled in equimolar concentration. The final library was purified using Agencourt AMPure XP Reagent beads (Beckman Coulter Life Sciences, Krefeld, Germany). The quality and integrity of the library were confirmed using the Agilent 2200 TapeStation with D1000 ScreenTapes (Agilent Technologies, Santa Clara, CA, USA). Sequences were generated at the Berlin Center for Genomics in Biodiversity Research (BeGenDiv) on the Illumina MiSeq platform (Illumina, San Diego, CA, USA) using v2 chemistry with 500 cycles (one run each for 210 and 236 laboratory samples with single and multiple amplicon products, respectively, and four runs for 672 wild mouse samples with multiple amplicon products). All sequencing raw data can be accessed through the BioProject PRJNA548431 in the NCBI Short Read Archive (SRA).

### Identification and quality screening of ASVs

We used the R packages dada2 [23] and MultiAmplicon [35] to filter, sort, merge, denoise and remove chimaeras for each run separately and for each amplicon. Additionally, we used the package DECONTAM [36] to remove contaminants and sequencing errors using “prevalence” and “frequency” methods (method = “combined”). We removed ASVs that have less than 1% prevalence, less than 0.005% relative abundance [37] and samples with less than a total sum of 100 reads. Filtering was done individually for each amplicon in the multi amplicon datasets and then all amplicon’s products were collated into one “phyloseq” object using the function “merge_phyloseq” implemented in the package “phyloseq”[38]. For the infection experiment, this resulted in 200 samples for the standard PCR and 218 samples for the microfluidic PCR. 619 samples for the microfluidic PCR on wild mouse samples were available for further sequence analysis after the same procedure.

### Taxonomic annotation of ASVs

A first taxonomic assignment of the resulting ASVs was performed based on the amplicon target with the assignTaxonomy function from dada2, using the RDP classifier [39]. 18S and 16S rRNA sequences were classified against SILVA 138.1 SSU Ref NR 99, 28S rRNA against SILVA 138.1 LSU Ref NR 99 databases [40] and ITS rRNA against the UNITE database [41]. Sequences and taxonomies from all other targeted regions which do not have a publicly available curated database were downloaded from NCBI, sequences with more than 5 degenerated bases and with lengths less than 300 bases were removed using RESCRIPt [42]. All databases were dereplicated using RESCRIPt [42]. For the present study, we focus on ASVs annotated as *Eimeria* in the amplicons targeting 18S rRNA and 28S rRNA genes.

To refine the taxonomic annotation, we constructed phylogenetic trees. To do so, we constructed alignments using the function AlignSeqs of the package DECIPHER [43] with “iterations = 20” and “refinements = 20”. We used maximum likelihood models and ModelFinder [44] for selecting the most appropriate evolution model, as implemented in iqtree2 [45]. The selected best-fitting model was TN + F + R2. We assessed branch support with an ultrafast bootstrap approximation (UFBoot) with 5000 replicates [46]. For the 18S rRNA gene, we included sequences of *Eimeria* species previously detected in house mice and other rodents [27], all 18S rRNA *Eimeria* ASVs recovered from this study and 18S rRNA sequences from the outgroup *Isospora* sp. ex *Talpa europaea*. Trees were visualised with iTOL version 6.7.4 [47].

The consensus tree resulted in poorly supported clades. Thus we constructed separate phylogenetic trees for all ASVs from each amplicon targeting the 18S rRNA gene, together with the *Eimeria* reference and outgroup sequences. We assigned a species name to each ASV that shared the most recent common ancestry with the reference sequences of *E. ferrisi*, *E. falciformis* and *E. vermiformis* in the rooted tree using the ape [48] and ggtree [49] packages. Bootstrap support values for each species clade were recorded in each tree, and indicate the reliability of species assignment for the respective amplicon’s ASVs (Additional file 2). One 18S rRNA ASV remained unassigned. We performed a BLAST search for each 28S rRNA ASV on the *E. falciformis* reference genome (ASM227181v1) [50]. Due to the lack of other reference sequences for the *Eimeria* 28S rRNA gene, we constructed a phylogenetic tree without those, including the eight 28S rRNA gene ASVs from the present study and the corresponding region of the *E. falciformis* genome to the 28S rRNA ASVs. For this tree, we found the substitution model TPM3u + F + R4 to provide the best fit and used it in phylogenetic reconstruction. Branch support was estimated after 5000 replicates. All alignments and phylogenetic trees with respective GenBank accession codes, including a tree with only reference sequences for comparison, are in Additional file 3.

To transfer taxonomic annotation to ASVs without phylogenetic clustering with reference sequence (i.e. when reference sequence was unavailable for the amplified region, as for 28S rRNA or when assignment is poorly supported in the phylogenetic analysis) we created a co-abundance network with all *Eimeria* ASVs allowing only significant Pearson correlation coefficients after adjusting for multiple testing using the Benjamini–Hochberg method.

### Normalisation

Normalisation contextualises the abundance of *Eimeria* sequences in the samples using the overall sequencing read count for other taxa (fungi and Eukaryotic parasites in case of 18S rRNA gene, also bacteria and archaea in case of 16S rRNA gene, as used in simultaneous microbiome PCRs on mice sampled in the natural environment). We evaluated different normalisation techniques by comparing correlation coefficients (Pearson’s rho) of sequencing-derived abundance with qPCR abundance. We tested the following normalisations: (1) total sum scaling (TSS), (2) trimmed mean by M-value (TMM) implemented in “microbial” [51], (3) centred log-ratio (CLR) as implemented in the “microbiome” package [52] and (4) rarefaction, as implemented in “phyloseq” [38].

We tested the significance of the differences between correlations with back-transformed averaged Fisher’s Zs [53] based on dependent groups with overlapping correlations, implemented with the package “cocor” [54]. We report *P*-values adjusted to multiple testing using the Benjamini–Hochberg method [55]. Based on the results of this comparison (Additional file 4: Table S1, Figures S1, S2), we decided to apply TSS normalisation for further analysis.

### *Eimeria* detection and quantification

We analysed the sensitivity and specificity of *Eimeria* detection against our reference method, qPCR, in experimentally infected laboratory mice. Sensitivity is defined here as the proportion of amplicon sequencing-positive samples in qPCR-positives (proportion of “true positives”). Specificity is the proportion of qPCR-negative samples in sequencing-negative samples (proportion of “true negatives”).

For the analysis of quantitative precision, we excluded samples with no *Eimeria* detection in qPCR or amplicon sequencing. We analysed whether the abundance of all ASVs annotated to the genus *Eimeria* within a sample correlates with *Eimeria* genome copies/ng DNA using Pearson correlation on log-transformed values.

We used linear mixed-effects models (lmms) to test whether the abundance of individual ASVs predicts qPCR-derived abundance (*Eimeria* genome copies). We include days post-infection (dpi) as a random effect to control for increasing DNA abundance during the infection. All lmms were performed with the package lme4 [56]. We tested the significance of random effects with the ranova function of the package “lmerTest” [57] and fixed effects with likelihood ratio tests (LRT; compared against an F-distribution) using the anova function to compare between the full model and a model with the predictor removed. The global goodness of fitness was examined as the LRT against intercept-only models. Model assumptions were investigated with diagnostic residual plots. The variance explained (R^2^) was calculated as in Nakagawa and Schielzeth [58].

### Analysis of *Eimeria* community structure

Adjusted prevalence and respective 95% confidence intervals were calculated with Sterne's exact method [59] implemented in the package “epiR” [60]. We analysed the variation in *Eimeria* spp. community composition by the marginal effects of locality of capture, year, host sex and host body mass index (BMI) and controlled for the effect of different sequencing depths by including a categorical factor indicating the sequencing run for each sample. For that, we used permutational multivariate analysis of variance (PERMANOVA) on a matrix of Jaccard similarity coefficient implemented with the package vegan [61], function adonis2, parameter by = “margin”. BMI was calculated as body weight divided by squared body length. We then tested the associations between BMI and *Eimeria* spp. infection status and abundance by applying mixed-effects linear models with either the infection status or the abundance of infection of *E. ferrisi*, *E. falciformis* and *E. vermiformis* as fixed effect and locality as random effect.

## Results

### High precision of amplicon sequencing-based quantification

**Fig. 1:**
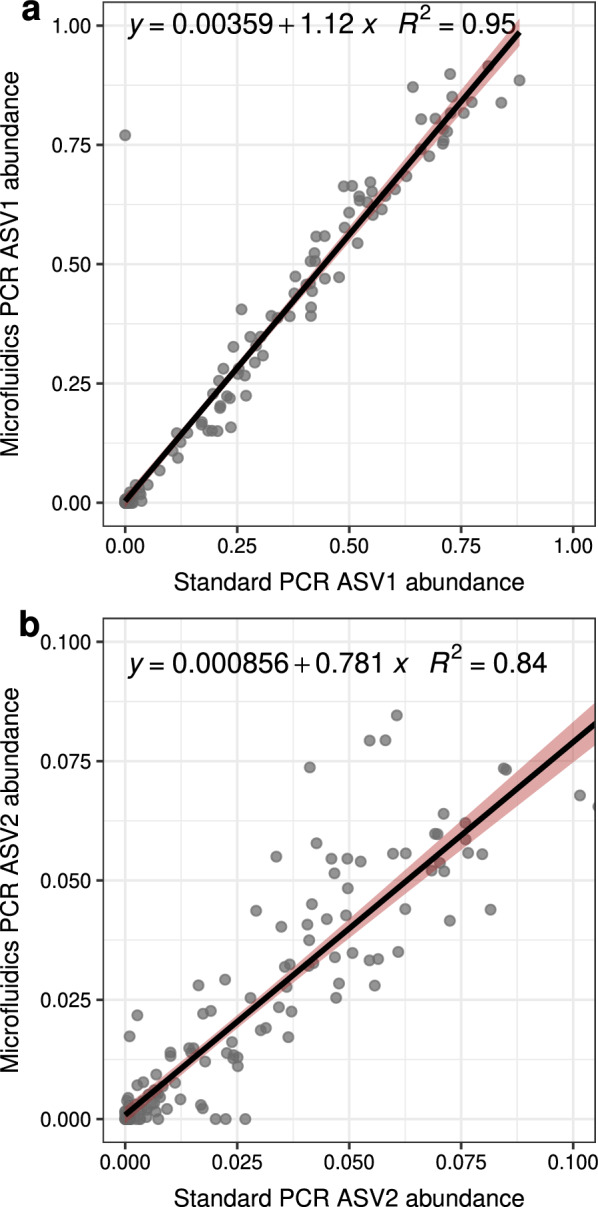
The same amplicon sequence variants (ASVs) annotated as *Eimeria*, sequenced from standard PCR and microfluidic PCR approaches are proportional. **a** ASV1, the most abundant ASV in both approaches, and **b** ASV2. Linear equation and coefficient of determination (*R*^2^) are reported for each curve. Regression lines are in black and shaded areas represent 95% confidence intervals. Abundance is expressed in relative abundance after total sum scaling

Sequencing of an 18S rRNA gene fragment amplified with a standard PCR approach in faecal samples of mice experimentally infected with *E. ferrisi* resulted in four ASVs in the genus *Eimeria*. Sequencing of the same amplicon, prepared with a microfluidic PCR protocol, resulted in two ASVs for *Eimeria* that correspond to the two most abundant ASVs produced with the standard PCR approach. The abundance of the two shared ASVs was highly correlated (ASV1: Pearson’s rho = 0.97, *df* = 180, *P* < 0.001; ASV2: Pearson’s rho = 0.92, *df* = 180, *P* < 0.001, Fig. 1). This indicates consistency in *Eimeria* quantification between standard and microfluidic PCR.

As “background amplification”, i.e. all amplified taxa, the standard PCR amplification targeting the 18S rRNA gene resulted in 57 sequenced taxa (four of these annotated as *Eimeria*), across 200 samples with a median sampling depth of 6245 reads and *Eimeria* ASV median sampling depth of 109 reads. The microfluidic PCR amplification was successful for 13 amplicons, resulting in 597 sequenced taxa (two of these annotated as *Eimeria*) across 218 samples with a median sampling depth of 7126 reads (17 median *Eimeria* sequencing depth). Out of these, the same amplicon targeted in the standard PCR amplification resulted in 43 sequenced taxa, including the two *Eimeria* ASVs, across 214 samples, with a median sampling depth of 2694 reads, and *Eimeria* ASV median sampling depth of 545 reads.

We tested different normalisations commonly applied in microbiome studies for their performance on our *Eimeria* ASV dataset. To do so we computed (Pearson) correlations between qPCR-based quantification (in genome copies per ng/DNA) and ASV abundance (summed over different ASVs). We compared the correlations for each normalised dataset against unnormalised data (sequencing read counts). Tested normalisations include TSS, TMM, CLR and rarefaction. The applied normalisations did not greatly impact the resulting correlations. However, TSS and rarefaction demonstrated the best agreement with qPCR-based abundance (Additional file 4: Figures S1, S2, Table S1). We thus decided to report data normalised with TSS.

**Fig. 2:**
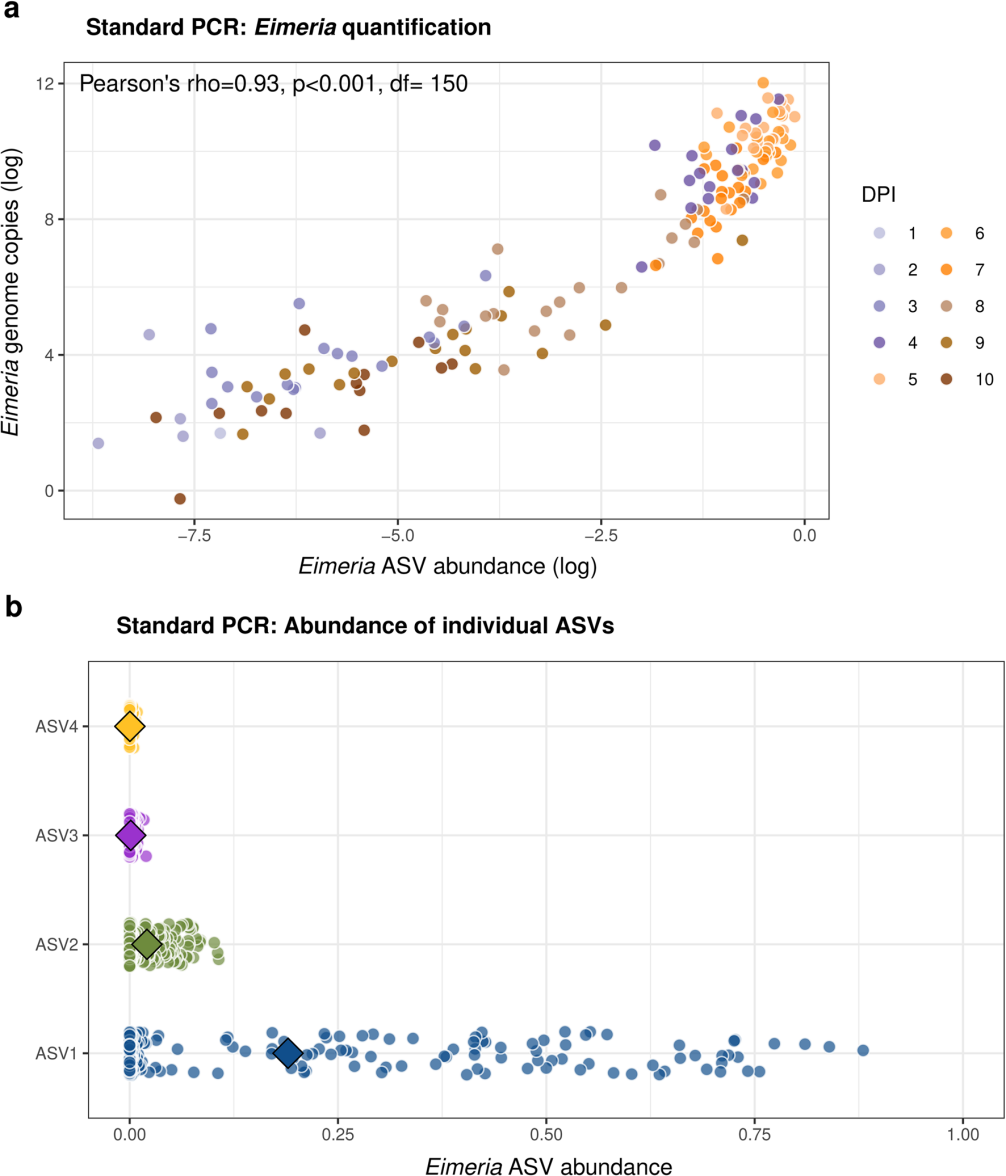
Precise *Eimeria* quantification with amplicon 18S rRNA gene fragment sequencing. **a** Relationship between *Eimeria* DNA measured with qPCR and ASVs from sequenced standard PCR. **b** Abundance distribution for each individual *Eimeria* ASV sequenced from standard PCR amplification. The mean for each ASV abundance is depicted as a diamond. dpi: days post-infection. Abundance is expressed as relative abundance after total sum scaling

Generally, *Eimeria* ASV abundance (summed of the two or four ASVs, respectively) was highly correlated with qPCR-derived abundance (genome copies/ng of DNA) for both sequencing approaches (standard PCR: rho = 0.93, *t* = 31.987, *df* = 150, *P* < 0.001; microfluidic PCR: rho = 0.89 *t* = 21.398, *df* = 126, *P* < 0.001; Fig. 2, Additional file 4: Figures S1, S2). We further tested the predictive value of each individual ASV abundance against qPCR-derived abundance using generalised linear mixed modelling (log-likelihood ratio tests (LRT) for standard PCR: *χ* = 82.4, *df* = 4, *P* < 0.001; microfluidic PCR: *χ* = 47.115, *df* = 2, *P* < 0.001, Table 1). ASV3 and ASV4 were not found to be predictive for qPCR-measured DNA abundance and are thus likely residual “noise” from sequencing errors. In contrast, ASV1 and ASV2 were significantly associated with qPCR-derived abundance for both amplification approaches. Overall, the variance explained by ASVs and dpi effects is very high (standard PCR conditional *R*^2^ = 0.89; microfluidic PCR conditional *R*^2^ = 0.79). ASVs explain much of the variance in the model (standard PCR marginal *R*^2^ = 0.66; microfluidic PCR marginal *R*^2^ = 0.52), even when controlling for dpi, which explains a large proportion of the variance in *Eimeria* abundance, as there is a clear progression of the infection through time (LRT for dpi as random effect in standard PCR: *χ* = 42.477, *df* = 1, *P* < 0.001; microfluidic PCR: *χ* = 8.694, *df* = 1, *P* = 0.003, Additional file 4: Figure S3). We can regard DNA quantification provided by amplicon sequencing as highly precise.

**Table 1:** The relationship of individual ASV abundance with qPCR-derived *Eimeria* genome copies/ng while controlling for days post-infection as a random effect

		Estimate	SE	*t*-value	*P*-value	*F*-value	*df*	*P*-value
(a)								
Standard PCR	Intercept	4.119	0.470	8.756	< 0.001*			
	ASV1	8.894	0.840	10.592	< 0.001*	34.665	1	< 0.001*
	ASV2	28.29	4.426	6.391	< 0.001*	4.567	1	< 0.001*
	ASV3	−12.064	26.970	−0.447	0.655	0.200	1	0.655
	ASV4	20.027	59.011	0.339	0.735	0.115	1	0.735
(b)								
Microfluidic PCR	Intercept	4.961	0.476	10.412	< 0.001*	–	–	–
	ASV1	6.389	0.785	8.134	< 0.001*	66.169	1	< 0.001*
	ASV2	27.096	5.555	4.878	< 0.001*	23.797	1	< 0.001*

(a) ASVs sequenced from standard amplification, *n* (samples) = 152, groups (dpi) = 10. (b) ASVs sequenced from microfluidic amplification, *n* (samples) = 128, groups (dpi) = 10. ASV abundance is the relative abundance after total sum scaling for each amplicon

SE, standard error; *Statistically significant

### Amplicon sequencing-based detection has imperfect specificity and sensitivity

We compared the sensitivity and specificity, of the merged ASVs annotated to *Eimeria* and individual ASVs. As for quantitative precision, we again compare standard amplification in microtiter plates with microfluidic amplification using the melting curve analysis in our qPCR as the “gold standard” for reference (Table 2). We found 13 false positives after standard PCR, but only three false positives with microfluidic PCR amplification. These have relatively low abundance when compared to the mean average of true-positive samples (mean ± standard deviation standard PCR: 0.001 ± 0.0009 vs 0.28 ± 0.27; microfluidic PCR: 0.26 ± 0.45 vs 0.35 ± 0.30). In contrast, we found 10 false negatives with standard PCR, but 35 with microfluidic PCR. This means that PCR amplification with standard methods had a relatively high sensitivity, but lower specificity while microfluidic amplification had inversely relatively low sensitivity but higher specificity. Both methods were imperfect in the detection of *Eimeria* infection compared against a highly sensitive qPCR.

**Table 2:** Detecting *Eimeria* sensitivity and specificity for sequencing of *Eimeria* ASVs produced with standard PCR and microfluidic PCR. *Eimeria* detection with qPCR (genome copies per nanograms of DNA) is the reference standard

	All *Eimeria* ASVs (%)	ASV1 (%)	ASV2 (%)
Sequenced with standard PCR			
Sensitivity	93.8	83.3	92.6
Specificity	64.9	89.2	64.9
Sequenced with microfluidic PCR			
Sensitivity	78.5	69.9	73.6
Specificity	94.1	98.0	94.1

All *Eimeria* ASVs: the sum of all ASVs annotated to *Eimeria* for each sample

### Amplicon sequencing distinguishes and quantifies three *Eimeria* species in wild house mice

We sequenced faecal samples of wild-caught mice using amplification of multiple amplicons on a microfluidic device. In the background, 34 amplicons were successfully amplified, resulting in 2880 taxa sequenced across 619 samples, with a mean sampling depth of 15056 reads. Out of these, we retrieved 37 *Eimeria* ASVs from 10 amplicons targeting the 18S rRNA gene and one targeting the 28S rRNA gene amplicon. *Eimeria* ASV median sampling depth was 273 reads. 163 mice (adjusted prevalence: 28.1%, 95% CI 23.5–33.1) showed an infection based on amplicon sequencing. The distribution of *Eimeria* ASVs per amplicon is represented in Additional file 5: Figure S4a. Assigning species annotation to ASVs will allow species quantification based on combined ASV abundance per species, but is highly dependent on the correctness of taxonomic annotation.

**Fig. 3:**
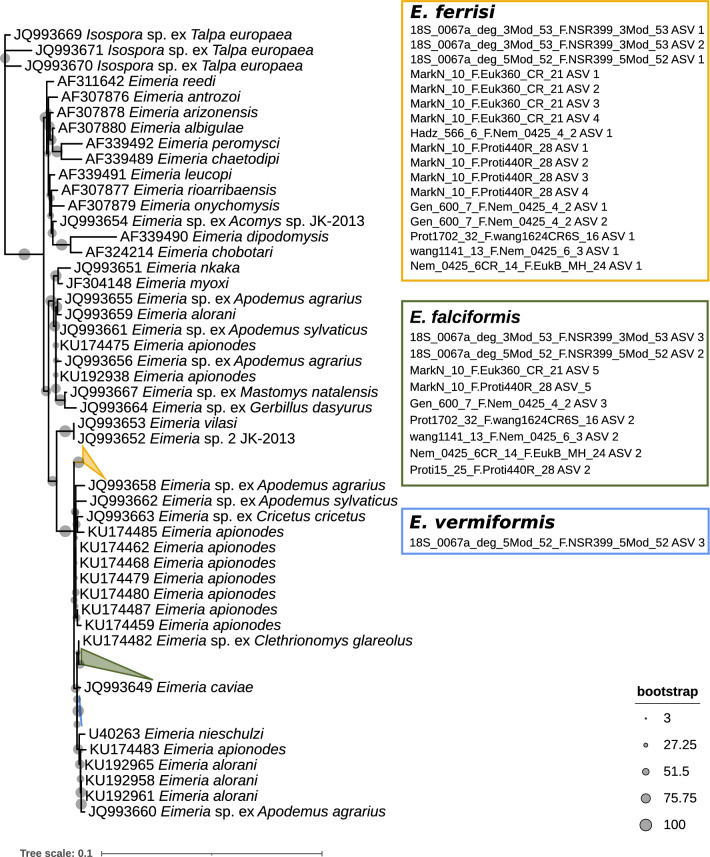
Phylogenetic analysis of *Eimeria* spp. for 18S rRNA gene reference sequences. Clades containing all reference sequences for *E. ferrisi* (yellow), *E. falciformis* (green) and *E. vermiformis* (blue) are collapsed and coloured. Coloured boxes represent the species assignment of ASVs based on phylogenetic clustering

**Fig. 4:**
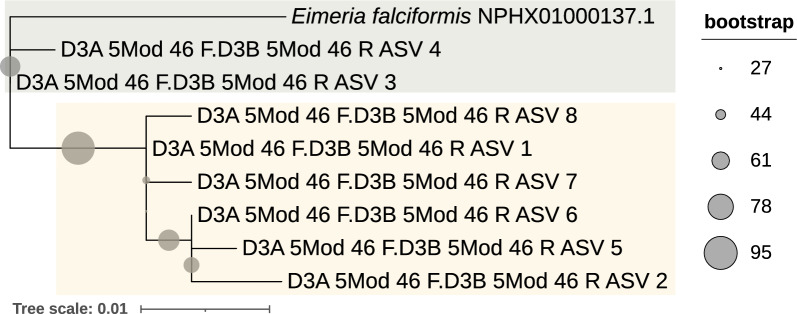
Phylogenetic analysis for *Eimeria* spp. based on the 28S rRNA gene. Shaded tips represent *E. ferrisi* (yellow) and *E. falciformis* (green) annotation according to ASV co-occurrence network

**Fig. 5:**
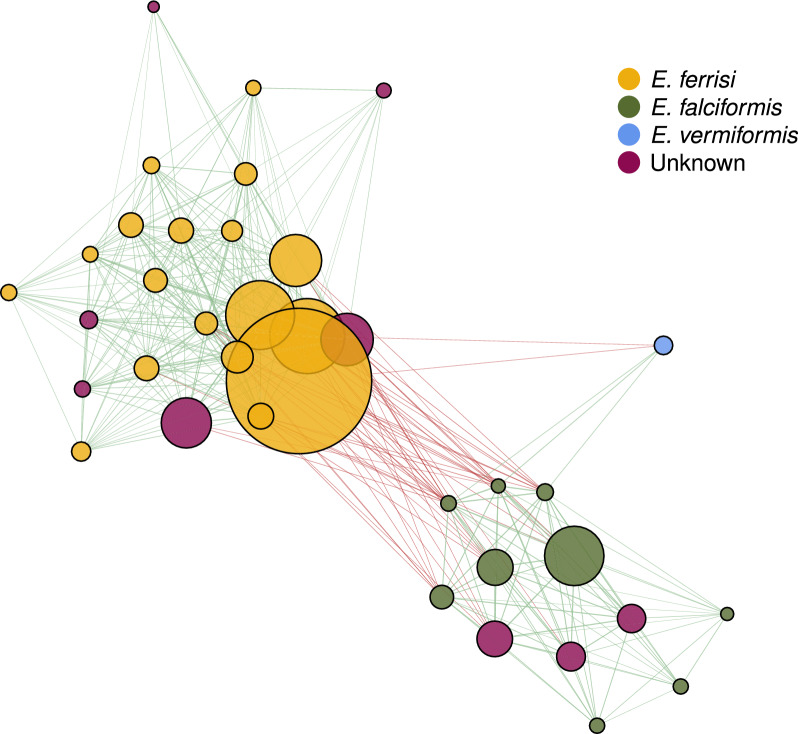
Co-occurrence network of ASVs annotated as three *Eimeria* species based on phylogenetic analysis. Nodes represent all ASVs annotated to *Eimeria* sequenced with a multiple amplicon approach. Node size reflects the frequency and relative abundance of each ASV: the relative abundance after total sum scaling within each amplicon, and summed across amplicons for each sample. Edges represent significant Pearson correlations after adjusting for multiple testing. Green edges mark positive correlations and red edges mark negative correlations

We thus improved taxonomic annotation, relative to established sequence-similarity-based methods, using a phylogenetic approach. This allowed us to resolve annotations for all but one of the 18S rRNA gene ASV with reference sequences of three *Eimeria* species known to infect house mice (Fig. 3). As there are no house mouse *Eimeria* reference sequences for the 28S rRNA gene, other than *E. falciformis,* phylogenetic analysis clustered the ASVs without allowing assignment of a species name at this point (Fig. 4). Two ASVs cluster with *Eimeria falciformis*, whereas six ASVs cluster together, suggesting the presence of two species. We then created a co-abundance network with all *Eimeria* ASVs showing that ASVs form three separate abundance clusters. Each of these abundance clusters contains ASVs annotated to one *Eimeria* species. Based on the position of the phylogenetically unassigned ASVs, it was then possible to assign them to a species: The unassigned ASVs from the 28S rRNA amplicon are divided between the *E. ferrisi* (six ASVs) and *E. falciformis* (two ASVs) clusters (Figs. 4, 5). The phylogenetically unassigned 18S rRNA gene ASV can be assigned to *E. falciformis* by this co-occurrence network. Overall, we were able to assign species to ASVs irrespective of the presence of reference sequences with a combination of phylogenetic analysis and a co-occurrence network. We identified 124 mice (adjusted prevalence: 19.5%, 95% CI 15.3–24.1%) infected with *E. ferrisi*, 58 mice (adjusted prevalence: 9.4%, 95% CI 7.2–11.9%) infected with *E. falciformis* and nine mice (adjusted prevalence: 1.4%, 95% CI 0.7–2.7%) infected with *E. vermiformis.* The distribution of *Eimeria* ASVs per species is presented in Additional file 5, Figure S4b.

### *Eimeria ferrisi* and *Eimeria falciformis* infection is associated with reduced body condition

To test the usability of amplicon sequencing-derived quantification, we then asked two questions: (i) whether the occurrence of different species is clustered within sampling localities and b) whether the body condition of mice is associated with *Eimeria* spp. infection. BMI was not available for 12 mice and was therefore removed for analysis. For these questions, we first analysed a multivariate response and found a strong effect of mice capture location on *Eimeria* spp. composition (*N* = 161, Permanova on Jaccard index: *R*^2^ = 0.47, pseudo-*F* = 1.340, *P* < 0.001, Additional file 5: Table S2), meaning that about half of the occurrences of infection with a particular species were explained by specific parasites circulating in a local population. This is an expected result from an epidemiological point of view, but also a confirmation of the detection accuracy of amplicon sequencing in a natural population. More strikingly, in a biological sense, we also found a smaller but still significant association of BMI (*R*^2^ = 0.02, pseudo-*F* = 3.752, *P* = 0.002, Additional file 5: Table S2) on *Eimeria* species composition, meaning that about 2% of the variation in *Eimeria* spp. composition was associated with the body condition of the mice. We controlled for the effect of different sampling depths due to samples being sequenced in different sequencing runs and found a small and not significant effect (*R*^2^ = 0.003, pseudo-*F* = 0.557, *P* = 0.849).

**Fig. 6:**
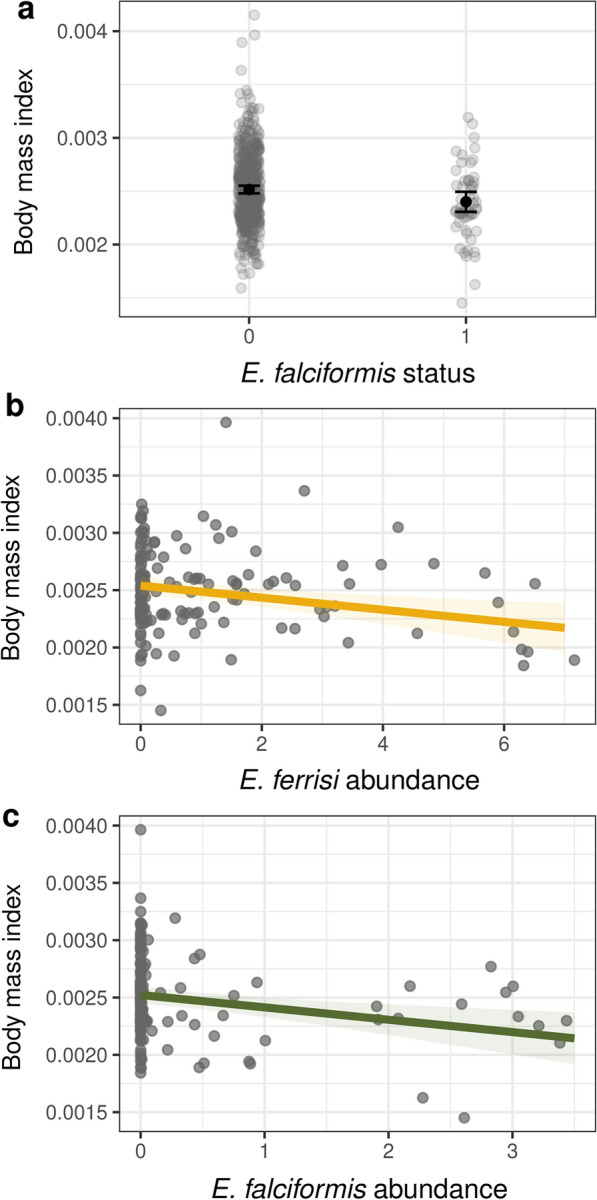
*Eimeria* infection and intensity predict poor body condition in mice. **a** *Eimeria falciformis* infection status, **b** *Eimeria ferrisi* abundance and **c** *Eimeria falciformis* abundance association with body mass index. Grey points represent the relationship (raw data) between body mass index and *Eimeria* spp. infection status or abundance. The mean predicted effect from a linear mixed model and associated 95% confidence intervals are represented as black points. Lines with respective coloured shades represent the predicted mean effect corresponding 95% confidence intervals. Abundance is the *Eimeria* spp. ASV relative abundance after total sum scaling within each amplicon, and summed across amplicons for each sample

We further investigated this finding with two models using BMI as a (univariate) response variable and (a) the infection status of *Eimeria* spp. as predictors (infected and uninfected, *N* = 607, LRT: *χ* = 10.064, *df* = 3, *P* = 0.018, Table 3a) and (b) the intensity of *Eimeria* spp. as predictors (*N* = 161, LRT: *χ* = 16.145, *df* = 3, *P* = 0.001, Table 3b). We controlled for location effects in these models (which were, as expected, significant: random effect for infection status model: LRT = 35.269, *df* = 1, *P* < 0.001, intensity model: LRT = 10.336, *df* = 1, *P* = 0.001). We found that animals infected with *E. falciformis* have a lower BMI when compared to uninfected animals (Fig. 6a, Table 3a), and the intensity of infection with *E. ferrisi* and *E. falciformis* abundance have a negative effect on BMI (Fig. 6b–c, Table 3b). Overall, the *Eimeria* spp. infection status and intensity of infection explained a substantial proportion of BMI variation (infection status model: marginal *R*^2^ = 0.02, conditional *R*^2^ = 0.18; infection intensity model: marginal *R*^2^ = 0.09, conditional *R*^2^ = 0.40). This demonstrates the usability of amplicon sequencing-derived detection and intensity estimates for biological questions.

**Table 3:** Generalised linear mixed effect models investigating (a) the infection status of *Eimeria* spp. (infected vs uninfected) and (b) the effects of *Eimeria* spp. abundance on the body mass index of house mice from a natural population

	Estimate	SE	*t*-value	*P*-value
(a) *Eimeria* spp. presence effects on body mass index, *N* = 607, localities = 171				
Intercept	0.00252	0.00002	132.173	< 0.001 *
*E. ferrisi*	−0.00001	0.00003	−0.423	0.672
***E. falciformis***	−0.00012	0.00005	−2.370	0.018 *
*E. vermiformis*	−0.00012	0.00011	−1.015	0.311
(b) *Eimeria* spp. infection intensity effects on body mass index, *N* = 163, localities = 67				
Intercept	0.00258	0.00004	62.904	< 0.001 *
***E. ferrisi***	−0.00005	0.00002	−3.132	0.002 *
***E. falciformis***	−0.00011	0.00003	−3.189	0.002 *
*E. vermiformis*	−0.00025	0.00033	−0.750	0.446

The location where mice were captured is included as a random effect. Abundance is the relative abundance after total sum scaling within each amplicon, and summed across amplicons for each sample

SE, standard error; *Statistically significant

## Discussion

Quantification of Coccidia based on DNA abundance is a suitable addition or alternative to classical coprological counts of transmission stages for Coccidia [15]. Assessment of DNA abundance using qPCR, however, does not allow scientists to distinguish different parasite species simultaneously. Sequencing of PCR products might provide an alternative to permit differential quantification of multiple species at once, but such methods are not well established and have seen relatively little use by parasitologists. The general applicability of amplification of universal marker regions and DNA sequencing makes this differential quantification of multiple species attractive for parasitologists working on any parasite shedding DNA into host faeces.

Hinsu et al.[5] reported higher sensitivity for single amplicon sequencing of the 18S rRNA gene than for species–specific qPCR in chicken sampled in farms and reported the detection of multiple *Eimeria* species. As discussed by the authors, an alternate explanation of a higher false-positive rate in the amplicon sequencing approach cannot be excluded. In this regard, using experimentally infected animals is more suitable to evaluate the detection and quantification of amplicon sequencing. In our previous work, we have shown that the qPCR accurately quantifies both *E. ferrisi* and *E. falciformis,* with about 0.6% variation in standard curves attributed to species differences [15]. This means our qPCR is equally applicable to the *Eimeria* species with high prevalence in house mice. Compared against qPCR, we show that *Eimeria* DNA can be accurately quantified using amplicon sequencing, while sensitivity and specificity of detection are—when used with caution, tolerably—imperfect. We tested two different amplification procedures and sequenced the resulting products. Both approaches resulted in identical ASVs, meaning that differences between the two methods can be partially attributed to differences in the depth of sequencing: sensitivity of detection is higher when sequencing more deeply, whereas specificity is higher at more shallow sequencing depth. This is analogous to classical coprological techniques, in which the assessed volume of the sample determines sensitivity. Because false positives are likely resulting from cross-contamination, common in amplicon sequencing [36, 62], the microfluidic amplification in closed chambers might provide additional specificity here. For both amplification schemes, however, the sensitivity and specificity of sequencing are considerably lower than the 100% reported in coprological trials with spiked oocysts [63]. There is, however, a lower limit of detection for coprological techniques [64], which are often hit in samples from natural populations. Specificity in the sense of avoiding false positives is hence dependent on amplification procedure and sequencing depth, but we argue that this is tolerable in applications on real-world samples. It is tolerable, because specificity in the sense of being able to detect and quantify different species differentially is more important. Additionally, quantitative precision in the comparison between the two amplification methods (~ 97%) and between both sequencing-based assessments and qPCR (~ 93%) was very high. To put this in perspective, this precision is higher than that between different flotation-counting techniques for chicken *Eimeria* [63].

We show that dedicated taxonomical annotation via phylogenetic analysis of ASVs can identify and differentiate *E. ferrisi*, *E falciformis* and *E. vermiformis* in mice from a natural population. These species are so closely related, that they are difficult to distinguish even with classical, non-quantitative sequencing approaches [26, 27]. It is hence necessary to use a modern approach to sequence processing, inferring probabilistically plausible sequence variants (ASVs), at a resolution of a single nucleotide difference [23] instead of operational taxonomic units (OTUs) collapsed at similarity thresholds. At single nucleotide resolution we detect intraspecific variability of even the relatively conserved 18S rRNA regions we amplified for *E. ferrisi* from our laboratory infection. We show that this variation is credible in two out of four ASVs and not arising from sequencing error or “chimeric” PCR artefacts [65], as abundance trajectories are consistent within individual mice. The two credible ASVs might result from intragenomic variation within single cells [66], or from variation in different merozoites, as suggested for *Sarcocystis* spp. [67, 68]. Interestingly, we detected the less abundant of the credible ASVs for *E. ferrisi* consistently earlier during controlled laboratory infection, meaning that the 18S rRNA gene variant might be associated with a genetic variant with faster development, such as those selected for precocious lines [69, 70]. By applying a phylogenetically guided taxonomic annotation in natural samples, we detect the three *Eimeria* species previously described in our study area [27] with multiple amplified fragments of the 18S rRNA gene. To unify taxonomic annotation across those amplicons, we use the correlation of abundance across different samples, a concept well established in metagenomic studies [71], but much simpler in its application to ASVs. In our wild-derived samples, the occurrence of infection with a particular *Eimeria* species in an individual host is explained to a large extent by the presence of the parasite species in the local host population. This is expected from local epidemiology, i.e. from the persistence of infections in the population [72, 73]. Controlling for this “locality effect”, we find the body condition of the mice to be negatively associated with the presence of *E. falciformis*, and *within infected mice*, negatively associated with *E. ferrisi* and *E. falciformis* abundance measured by amplicon (DNA) sequencing. Such correlation of body condition with *Eimeria* infection has been reported in other mammals [74, 75], but we were unable to detect it in our system with the quantification of oocysts or DNA in tissue [30]. It is plausible that *Eimeria* infection causes poor body condition considering the noticeable weight loss usually seen in laboratory infection [33]. The observed nonsignificant effect of *E. ferrisi* infection, *E. vermiformis* infection and intensity on body condition might be due to poor statistical power (small effect and low prevalence, particularly for *E. vermiformis*) or due to the difficulties with the accuracy (sensitivity and specificity) of detection (particularly for *E. ferrisi* in the qualitative analysis). It was, however, possible to detect a negative effect of *E. ferrisi* intensity on BMI, this effect was smaller than that of *E. falciformis* but still significant. The negative association between body condition and the presence of *E. falciformis,* despite its lower prevalence than *E. ferrisi,* suggests a stronger effect than the latter that also fits observations from laboratory infection [32]. This demonstrates the potential of amplicon sequencing to address epidemiological and ecological questions previously hard to address in wildlife systems. We recommend that such effects are best analysed in a quantitative way relating them to infection intensity derived from sequencing counts.

Amplicon sequencing generates sparse (meaning many samples without detection of a particular taxon) and proportional (meaning that taxa abundance estimates impact each other, as they are obtained as a fraction of the overall sequencing reads) [76–78]. This results in a negative correlation bias, as an increase of a taxon is accompanied by a decrease in the remaining taxa. Normalisation techniques accounting for this [22, 78] are increasingly important if the analysed taxa make up a large proportion of the overall sequencing reads. We see this in our results, as the choice of normalisation is more important for quantitative precision in the less deeply sequenced microfluidic amplification, in which *Eimeria* sequences make up a larger proportion compared to the more deeply sequenced classical amplification. More importantly, suitable selection of the PCR primers to allow both good on-target (here: *Eimeria*) and wide taxonomically off-target reference for relative quantification, and sequencing at a suitable sequencing depth help to avoid such challenges. Many factors can affect the results of next-generation sequencing studies, including experimental design and analytical methods; therefore, meticulous consideration of the bioinformatics and statistical methodology is crucial. The optimal primer choice and sequencing depth are almost impossible to establish a priori, but we interpret our results as an encouragement to dare conducting such experiments in Coccidia and other parasites, as results will be robust across a wide range of parameters and can be corrected statistically for imperfect choices of molecular methods.

## Conclusion

Amplicon sequencing provides a unified, scalable methodology to study parasites (or generally, eukaryotic symbionts), the bacterial microbiome, and hosts (e.g. if specific amplicons are used for simultaneous genotyping). “Universal” primer pairs, mostly targeting the 18S rRNA gene, are available or can be designed [17]. More work on amplicons sequencing is needed to evaluate how different primer pairs amplify and quantify Coccidia, other parasites and eukaryotic symbionts in faecal samples from mammals and other hosts. We have shown here that amplicon sequencing can differentially quantify *Eimeria* over a wide range of amplicon (primer pair) choices. This sequencing-based DNA quantification of Coccidia has good precision relative to qPCR-based quantification, and there is no reason to suspect this would be different for other parasites. We have previously shown that DNA-based quantification and classical coprological counts of transmission stages are complementary [15]. DNA intensity should be evaluated with respect to othe0.00011r biologically relevant measures in more host–parasite systems and amplicons sequencing is a suitable quantitative tool for this.

## Supplementary Information

**Additional file 1: **Primer pairs used for multi-amplicon sequencing in the experimental (laboratory) and natural population datasets.

**Additional file 2: **Sequence alignments and respective phylogenetic trees.

**Additional file 3: ***Eimeria* taxonomic annotation to species level based on phylogenetic analysis. A phylogenetic tree was produced for all *Eimeria* ASVs from each amplicon (10 amplicons targeting the 18S rRNA gene), reference sequences for *Eimeria spp* of house mice and other rodents. Bootstrap_MRCA_clade: Bootstrap values from the clade of each species (reference species sharing the most recent common ancestor) retrieved from the respective amplicon phylogenetic tree.

**Additional file 4: Table S1.** Analysing the effects of normalisation techniques in the quantification of *Eimeria*. Comparison of two overlapping Pearson correlations based on dependent groups. Reference correlation is between *Eimeria* genome copies and *Eimeria* merged ASV abundance not normalised. rarefaction: random resampling of the OTU table to the minimum library depth. In green are the significantly improved and in red are significantly worsened correlations in relation to the overlapping correlation between *Eimeria* genome copies per gram of faeces and *Eimeria* ASV counts. P-adj: *P*-values corrected for multiple testing with Benjamini–Hochberg. **Figure S1.** Comparison of normalisation methods for quantification of *Eimeria* with amplicon sequencing (18S rRNA gene) using a standard PCR. dpi = days post-infection, *df* = degrees of freedom. **Figure S2.** Comparison of normalisation methods for quantification of *Eimeria* with amplicon sequencing (18S rRNA gene) using microfluidic PCR. dpi = days post-infection, *df* = degrees of freedom. **Figure S3.** The different amplified *Eimeria* ASVs consistently reflect infection dynamics. *Eimeria* ASV abundance throughout days post-infection measured as (a) ASV1 sequenced from standard PCR amplification; (b) ASV2 sequenced from standard PCR amplification; (c) ASV1 sequenced from microfluidic PCR amplification; (d) ASV1 sequenced from microfluidic PCR amplification. Abundance is measured as relative abundance after a total sum scaling normalisation, within the respective amplicon. Each dot represents one sample, samples from the same individual are connected with grey lines. The mean for each day post-infection is depicted as a diamond. Colours represent the different days post-infection. Note the difference in the scale of the y-axis in (a) and (c) vs (b) and (d).

**Additional file 5: Table S2.** Summary results of PERMANOVA based on the overall variation of *Eimeria* spp. composition, using the Jaccard similarity coefficient. Significant predictors are in bold. **Figure S4: **The distribution of *Eimeria* ASV abundance in mice sampled in the natural environment. (a) ASVs coloured by amplicon; (b) ASVs coloured by *Eimeria* species. Samples are ordered in both axes based on the first axis of a principal coordinate analysis with Bray–Curtis dissimilarities. Abundance is the relative abundance after total sum scaling within each amplicon, and summed across amplicons for each sample.

## Data Availability

The datasets supporting the conclusions of this article are in https://github.com/ferreira-scm/Eimeria_AmpSeq.git. All sequencing raw data can be accessed through the BioProject PRJNA548431 in the NCBI Short Read Archive (SRA).

## Abbreviations

ASVs: Amplified sequence variants
*COI*: Cytochrome *c* oxidase
CLR: Centred log-ratio
OTU: Operational taxonomic units
PERMANOVA: Permutational multivariate analysis of variance
PCR: Polymerase chain reaction
qPCR: Quantitative polymerase chain reaction
rRNA: Ribosomal RNA
TSS: Total sum scaling
