# Supplementary material for: Amplicon sequencing allows differential quantification of closely related parasite species: an example from rodent Coccidia (Eimeria)
Source: Parasit Vectors. 2023 Jun 17;16:204. doi: 10.1186/s13071-023-05800-6 (PMC10276917; doi:10.1186/s13071-023-05800-6)

**Additional file 4:**

**Table S1.** Analysing the effects of normalisation techniques in the quantification of *Eimeria*. Comparison of two overlapping Pearson correlations based on dependent groups. Reference correlation is between *Eimeria* genome copies and *Eimeria* merged ASV abundance not normalised. rarefaction: random resampling of the OTU table to the minimum library depth. In green are the significantly improved and in red are significantly worsened correlations in relation to the overlapping correlation between *Eimeria* genome copies per gram of faeces and *Eimeria* ASV counts. P-adj: P-values corrected for multiple testing with Benjamini-Hochberg.

|  | Standard PCR | Microfluidics PCR |
| --- | --- | --- |
| Total sum scaling | Rho difference= -0.0040  df= 152  z= -0.986  p-adj= 0.376 | Rho difference= -0.0437  df= 128  z= -2.833,  p-adj= 0.009 |
| Trimmed mean by M-values | Rho difference= -0.0055  df= 152  z= -1.625  p-adj= 0.208 | Rho difference= 0.0000  df= 128  z= 0.070,  p-adj= 0.945 |
| Centered log ratio | Rho difference= 0.0625  df= 152  z= 5.203,  p-adj<0.001 | Rho difference= -0.0065  df= 128  z= -0.350  p-adj= 0.945 |
| Rarefied read counts | Rho difference= -0.0092  df= 118  z= -0.886,  p-adj= 0.376 | Rho difference= -0.0934  df= 110  z= -3.322  p-adj= 0.004 |

**Figure S1.** Comparison of normalisation methods for quantification of *Eimeria* with amplicon sequencing (18S rRNA gene) using a standard PCR. dpi= days post infection, df= degree of freedom.


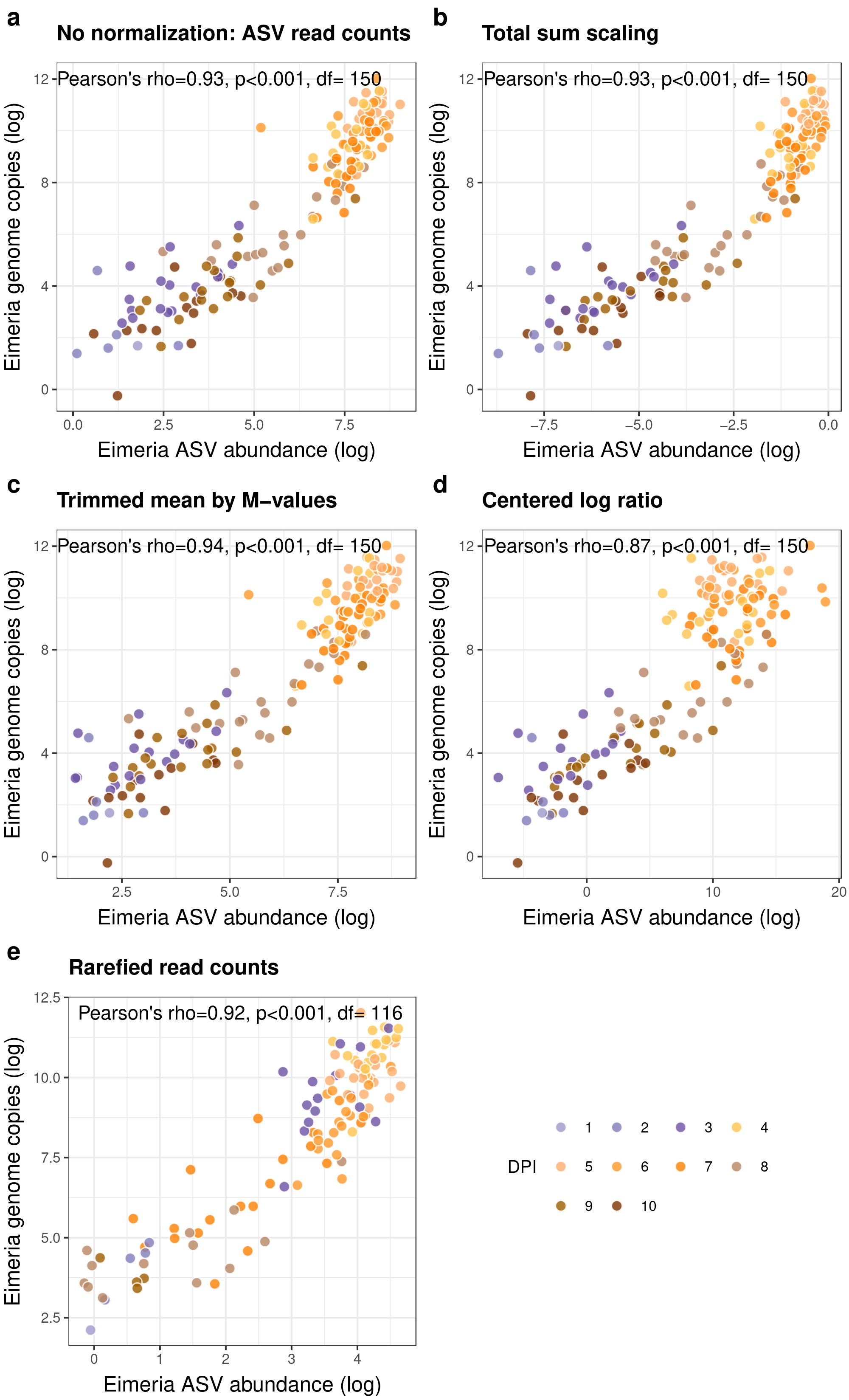


**Figure S2.** Comparison of normalisation methods for quantification of *Eimeria* with amplicon sequencing (18S rRNA gene) using microfluidics PCR. dpi= days post infection, df= degree of freedom.


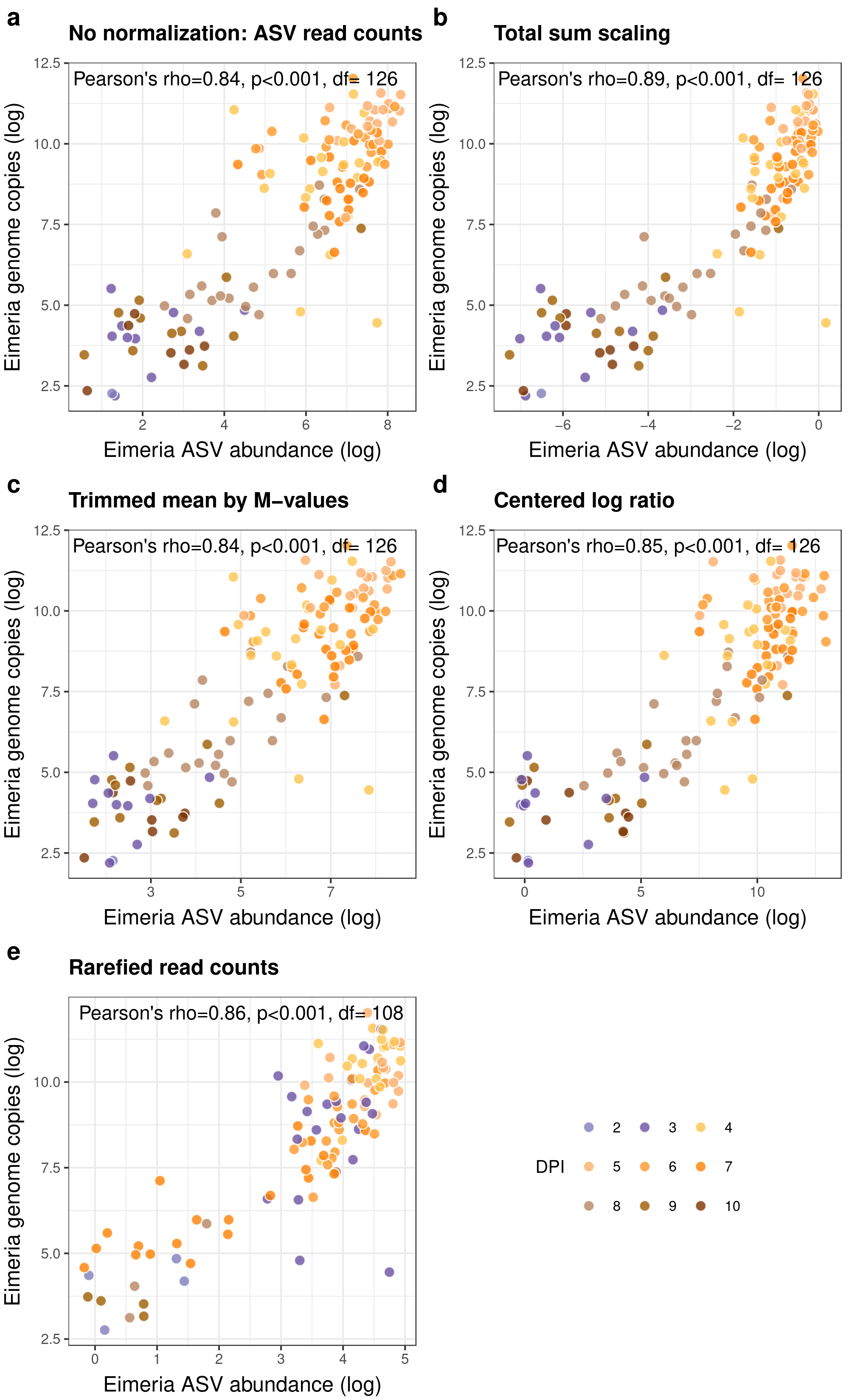


**Figure S3.** The different amplified *Eimeria* ASVs consistently reflect infection dynamics. *Eimeria* ASV abundances throughout days post infection measured as a) ASV1 sequenced from standard PCR amplification; b) ASV2 sequenced from standard PCR amplification; c) ASV1 sequenced from microfluidics PCR amplification; d) ASV1 sequenced from microfluidics PCR amplification. Abundances are measured as relative abundances after a total sum scaling normalisation, within the respective amplicon. Each dot represents one sample, samples from the same individual are connected with gray lines. The mean for each day post infection is depicted as a diamond. Colours represent the different days post infection. Note the difference in the scale of the y-axis in a) and c) vs b) and d).


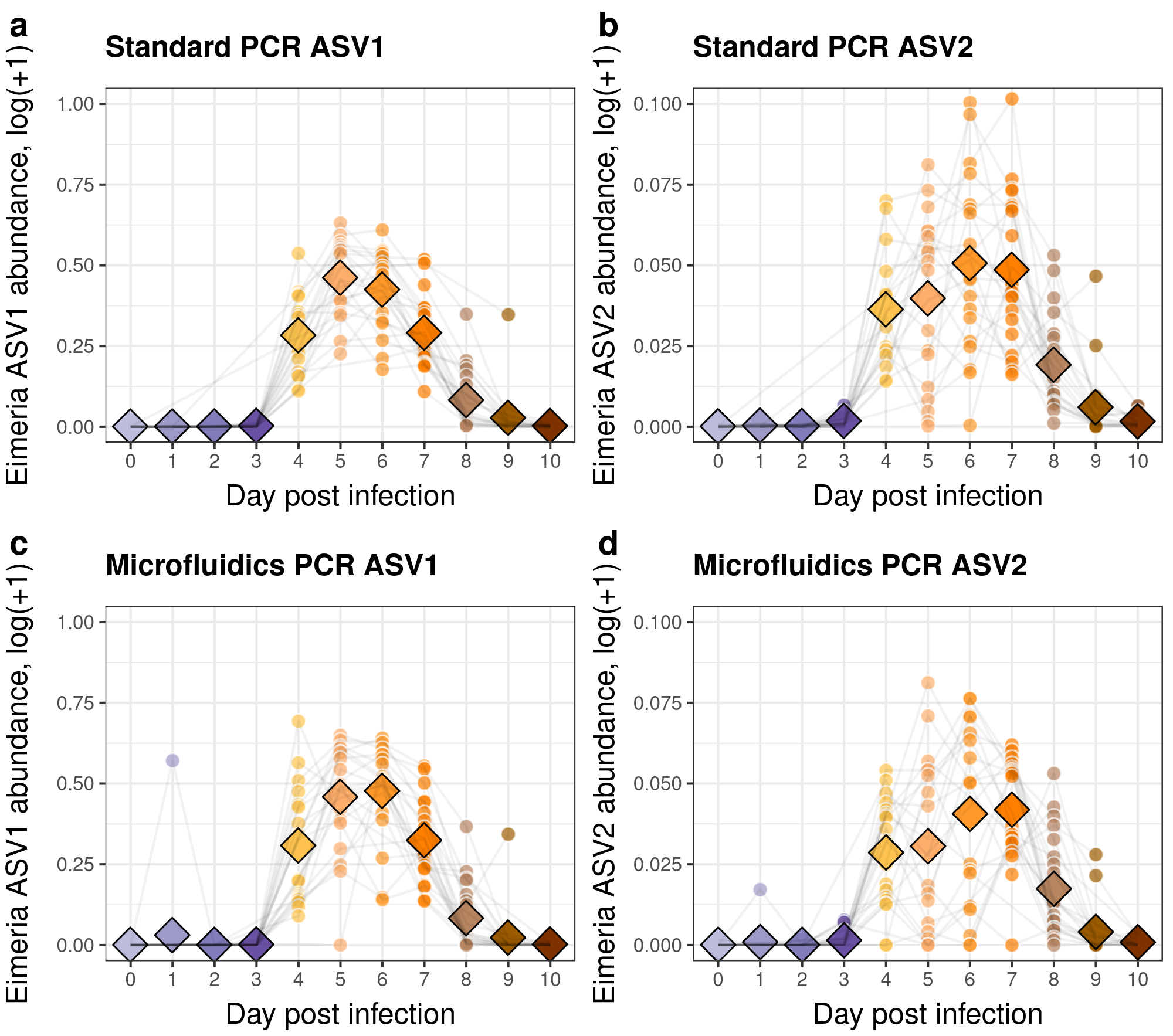

Supplement: Supplementary file 4 — Additional file 4: Table S1. Analysing the effects of normalisation techniques in the quantification of Eimeria. Comparison of two overlapping Pearson correlations based on dependent groups. Reference correlation is between Eimeria genome copies and Eimeria merged ASV abundance not normalised. rarefaction: random resampling of the OTU table to the minimum library depth. In green are the significantly improved and in red are significantly worsened correlations in relation to the overlapping correlation between Eimeria genome copies per gram of faeces and Eimeria ASV counts. P-adj: P-values corrected for multiple testing with Benjamini–Hochberg. Figure S1. Comparison of normalisation methods for quantification of Eimeria with amplicon sequencing (18S rRNA gene) using a standard PCR. dpi = days post-infection, df = degrees of freedom. Figure S2. Comparison of normalisation methods for quantification of Eimeria with amplicon sequencing (18S rRNA gene) using microfluidic PCR. dpi = days post-infection, df = degrees of freedom. Figure S3. The different amplified Eimeria ASVs consistently reflect infection dynamics. Eimeria ASV abundance throughout days post-infection measured as (a) ASV1 sequenced from standard PCR amplification; (b) ASV2 sequenced from standard PCR amplification; (c) ASV1 sequenced from microfluidic PCR amplification; (d) ASV1 sequenced from microfluidic PCR amplification. Abundance is measured as relative abundance after a total sum scaling normalisation, within the respective amplicon. Each dot represents one sample, samples from the same individual are connected with grey lines. The mean for each day post-infection is depicted as a diamond. Colours represent the different days post-infection. Note the difference in the scale of the y-axis in (a) and (c) vs (b) and (d). [file 13071_2023_5800_MOESM4_ESM.docx]
