# Supplementary material for: Amplicon sequencing allows differential quantification of closely related parasite species: an example from rodent Coccidia (Eimeria)
Source: Parasit Vectors. 2023 Jun 17;16:204. doi: 10.1186/s13071-023-05800-6 (PMC10276917; doi:10.1186/s13071-023-05800-6)

**Additional file 5:**

**Table S2.** Summary results of PERMANOVA based on the overall variation of *Eimeria* spp. composition, using Jaccard similarity coefficient. Significant predictors are in bold.

|  | **DF** | **R**2 | **F** | **p** |
| --- | --- | --- | --- | --- |
| **Locality** | **65** | **0.472** | **1.340** | **0.001** |
| Year | 2 | 0.012 | 1.109 | 0.351 |
| Sex | 1 | 0.009 | 1.642 | 0.101 |
| **BMI** | **1** | **0.020** | **3.752** | **0.002** |
| Sequencing Run | 1 | 0.003 | 0.557 | 0.849 |
| Residual | 89 | 0.482 | - | - |
| Total | 160 | 1 | - | - |

**Figure S4:** The distribution of *Eimeria* ASVs abundance in mice sampled in the natural environment. a) ASVs coloured by amplicon; b) ASVs coloured by *Eimeria* species. Samples are ordered in both axes based on the first axis of a principal coordinate analysis with Bray-Curtis dissimilarities. Abundances are the relative abundances after total sum scaling within each amplicon, and summed across amplicons for each sample.


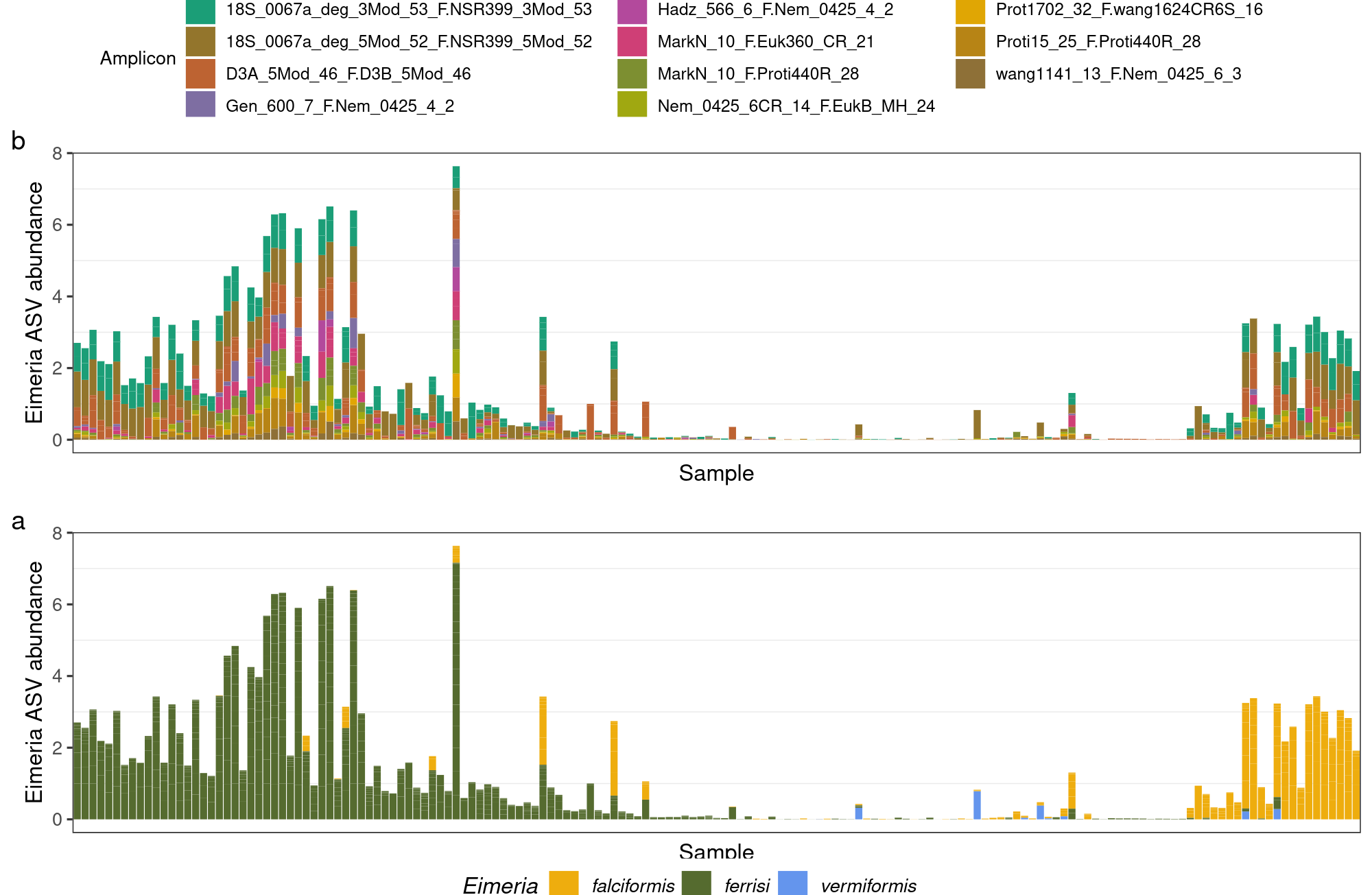

Supplement: Supplementary file 5 — Additional file 5: Table S2. Summary results of PERMANOVA based on the overall variation of Eimeria spp. composition, using the Jaccard similarity coefficient. Significant predictors are in bold. Figure S4: The distribution of Eimeria ASV abundance in mice sampled in the natural environment. (a) ASVs coloured by amplicon; (b) ASVs coloured by Eimeria species. Samples are ordered in both axes based on the first axis of a principal coordinate analysis with Bray–Curtis dissimilarities. Abundance is the relative abundance after total sum scaling within each amplicon, and summed across amplicons for each sample. [file 13071_2023_5800_MOESM5_ESM.docx]
